# Cytotoxicity of ZnO Nanowire Arrays on Excitable Cells

**DOI:** 10.3390/nano7040080

**Published:** 2017-04-07

**Authors:** Yongchen Wang, Yu Wu, Farhan Quadri, Jordan D. Prox, Liang Guo

**Affiliations:** 1 Department of Biomedical Engineering, The Ohio State University, Columbus, OH 43210, USA; wang.4896@osu.edu; 2 Department of Electrical and Computer Engineering, The Ohio State University, Columbus, OH 43210, USA; wu.2233@osu.edu; 3 Department of Food, Agricultural and Biological Engineering, The Ohio State University, Columbus, OH 43210, USA; quadri.8@osu.edu; 4 Biomedical Sciences Graduate Program, The Ohio State University, Columbus, OH 43210, USA; prox.6@osu.edu; 5 Department of Neuroscience, The Ohio State University, Columbus, OH 43210, USA

**Keywords:** ZnO nanowire arrays, cytotoxicity, excitable cells

## Abstract

Zinc oxide (ZnO) nanowires have been widely studied for their applications in electronics, optics, and catalysts. Their semiconducting, piezoelectric, fluorescent, and antibacterial properties have also attracted broad interest in their biomedical applications. Thus, it is imperative to evaluate the biosafety of ZnO nanowires and their biological effects. In this study, the cellular level biological effects of ZnO nanowire arrays are specifically tested on three types of excitable cells, including NG108-15 neuronal cell line, HL-1 cardiac muscle cell line, and neonatal rat cardiomyocytes. Vertically aligned and densely packed ZnO nanowire arrays are synthesized using a solution-based method and used as a substrate for cell culture. The metabolism levels of all three types of cells cultured on ZnO nanowire arrays are studied using the 3-(4,5-dimethyl-2-thiazolyl)-2,5-diphenyl-2*H*-tetrazolium bromide (MTT) assays of a full factorial design. Under the studied settings, the results show statistically significant inhibitory effects of ZnO nanowire arrays on the metabolism of NG108-15 and HL-1 cells in comparison to gold, glass, and polystyrene substrates, and on the metabolism of cardiomyocytes in comparison to gold substrate.

## 1. Introduction

ZnO nanowires have attracted great research and industrial interest in their applications in photocatalysts, field-effect transistors, solar cells, and generators, for their semiconducting, piezoelectric, and optical properties [1,2,3,4]. These properties along with their nanomaterial attributes have also stimulated interest in their biomedical applications such as bioimaging, biosensing, antibacterial treatment, and nanogenerators powering wearable and implantable medical devices [2,5,6,7]. Such interest involves the handling and/or implantation of ZnO nanowires and thus calls for scrutiny on the biosafety of ZnO nanowires.

The biocompatibility or cytotoxicity of nanomaterials is shape-dependent [8]. For example, ZnO rod-shaped nanoparticles induced lower cytotoxicity on ANA-1 macrophage cells in comparison to ZnO spherical nanoparticles [9]. Despite numerous studies on the biosafety of ZnO nanoparticles, there have been only a few studies covering the biocompatibility of ZnO nanowires. ZnO nanowires or nanorods were shown to be biocompatible to HeLa cells and have a quantity-dependent toxicity on L929 fibroblast cells [10,11]. High aspect ratio ZnO nanowires also showed toxicity on human monocyte-derived macrophages [12]. Among the limited number of studies, little work has been done on the biological effects of ZnO nanowires on excitable cells, including neuronal cells or cardiac muscle cells which are both important classes of cells relevant to the application of ZnO nanowires. In addition, intraperitoneally administered ZnO nanoparticles were shown to cause cognitive impairment in rats [13], and accumulate in the heart of ICR mice [14]. Therefore, it is imperative to study the biological effects of ZnO nanowires on excitable cells.

As the cytotoxicity of nanomaterials also depends on the cell type [15], in this work, we assessed the biocompatibility of ZnO nanowire arrays to three types of excitable cells, including NG108-15 neuronal cell line, HL-1 cardiac muscle cell line, and primary neonatal rat cardiomyocytes. Specifically, we tested the metabolism of the cells cultured on ZnO nanowire arrays. We selected NG108-15 neuronal cell line as a neuronal cell model [16], HL-1 cardiac muscle cell line as a cardiomyocyte cell model [17], and neonatal rat cardiomyocytes as a primary cardiomyocyte cell model. Our data showed that after one day in culture ZnO nanowire arrays caused inhibitory effects on NG108-15 and HL-1 cells in comparison to gold, glass, and polystyrene substrates, and inhibitory effects on cardiomyocytes in comparison to gold substrate.

## 2. Results

### 2.1. Synthesis of ZnO Nanowire Arrays

ZnO nanowire arrays were grown on gold (Au)-coated cover glasses, resulting in a thin and uniform grayish layer over the Au coating. By examining the weight differences of six coated cover glasses before and after ZnO nanowire growth using an analytical balance, we found that on average, approximately 4 mg of ZnO nanowires were grown on each cover glass after 24 h of growth.

### 2.2. Scanning Electron Microscopy (SEM) Images of ZnO Nanowire Arrays

**Figure 1:**
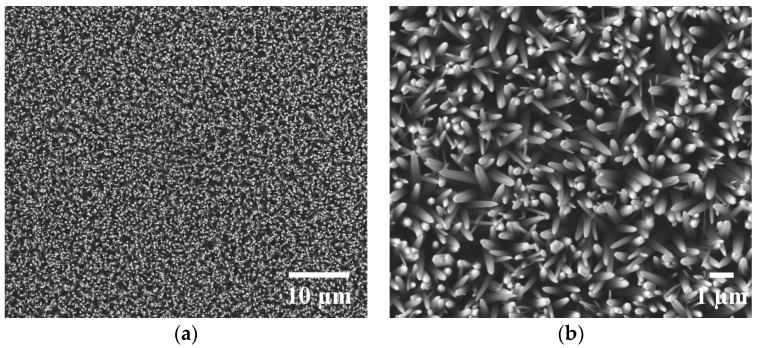
Scanning electron microscopy (SEM) images of zinc oxide (ZnO) nanowire arrays: (**a**) SEM image at a magnification of 2500×; (**b**) SEM image at a magnification of 10,000×.

The surface feature of the as-synthesized ZnO nanowire arrays was imaged with scanning electron microscopy (SEM) (Figure 1). The ZnO nanowires were uniformly distributed, vertically aligned, and densely packed into arrays as shown in Figure 1a (2500× magnification). The nanowires showed a U-shaped tip with the majority measuring about 300 nm in width (Figure 1b, 10,000× magnification).

### 2.3. X-ray Photoelectron Spectroscopy (XPS) Spectra of ZnO Nanowire Arrays

**Figure 2:**
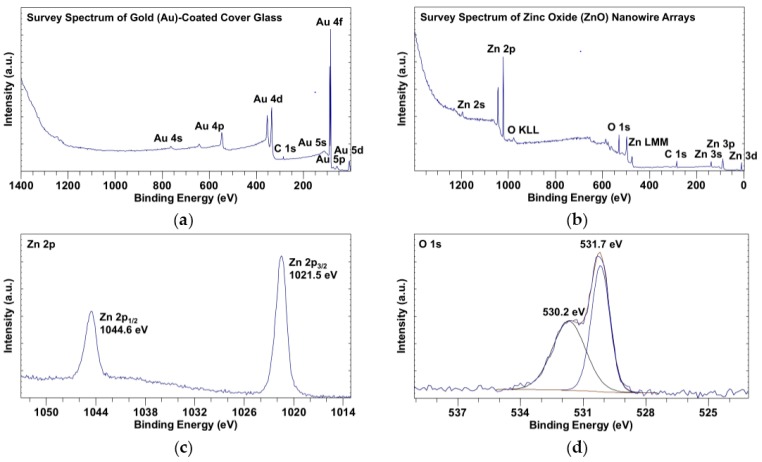
X-ray photoelectron spectroscopy (XPS) spectra of Au-coated cover glass and ZnO nanowire arrays: (**a**) survey spectrum of Au-coated cover glass; (**b**) survey spectrum of ZnO nanowire arrays; (**c**) Zn 2p spectrum of ZnO nanowire arrays; (**d**) deconvoluted O 1s spectrum of ZnO nanowire arrays.

The surface chemistry of ZnO nanowire arrays was analyzed using X-ray photoelectron spectroscopy (XPS), with Au-coated cover glass serving as a control. All the peak positions with respect to binding energy were calibrated by fixing the peak of adventitious carbon 1s (C 1s) to 285 eV [18,19]. As shown in the survey spectra of Au-coated cover glass (Figure 2a) and ZnO nanowire arrays (Figure 2b), there was no detectable level of elements other than gold and adventitious carbon on the surface of coated cover glass, while after ZnO nanowire growth, the surface of ZnO nanowire arrays mainly contained zinc, oxygen, and adventitious carbon. In the zinc 2p (Zn 2p) spectrum (Figure 2c), the doublet peaks of Zn 2p_1/2_ at 1044.6 eV and of Zn 2p_3/2_ at 1021.5 eV were characteristic of a hexagonal structure of nanowires [1,20]. In the deconvoluted oxygen 1s (O 1s) spectrum (Figure 2d), the peak of O 1s was fitted to two components at 530.2 eV and 531.7 eV. The component at 530.2 eV was attributed to the oxygen from ZnO, and the component at 531.7 eV was attributed to the chemisorbed oxygen [21,22]. As shown in Table 1, the ratio of percentage atomic concentrations of Zn 2p to O 1s (530.2 eV) was approximately 1 (19.51%:20.92%), which matched the stoichiometric ratio of Zn to O in ZnO.

### 2.4. MTT Assay of NG108-15 Cells

The significance level of all statistical analyses was pre-set as α = 0.05, so a *p*-value smaller than 0.05 indicated statistical significance. As shown in Table 2, MTT assays for all three cell types were conducted following a full factorial 2 × 4 design of two independent factors, substrate material (factor A) and existence of cells (factor B). Factor A was a discrete variable of four levels (ZnO nanowire arrays (ZnO), Au-coated cover glasses (Au), uncoated cover glasses (GL), and cell culture-treated polystyrene multi-well culture plates (PS)); Factor B was a discrete variable of two levels (with cells (cells) and with culture medium only (medium)). Thus, there were a total of eight combinations (ZnO + cells, Au + cells, GL + cells, PS + cells, ZnO + medium, Au + medium, GL + medium, and PS + medium). The metabolism of cells cultured on a specific substrate material was studied by subtracting the absorbance of material + medium from the absorbance of corresponding material + cells. All metabolism of cells was normalized to that of the cells cultured on PS, which served as the baseline.

**Figure 3:**
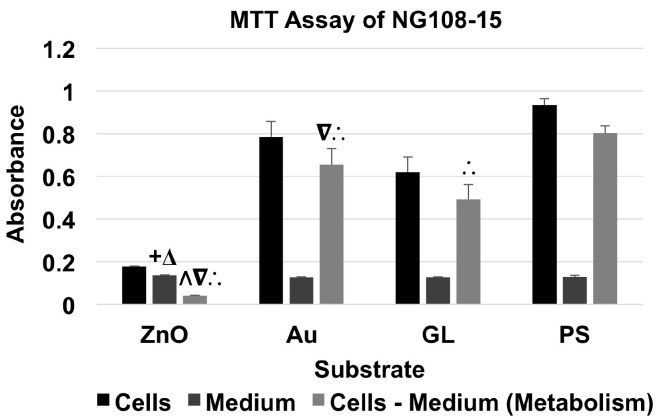
MTT assay of NG108-15 cells showed the absorbance of material + NG108-15 cells and material + medium, and the absorbance for the metabolism of NG108-15 cells (data were presented as mean + standard deviation; standard deviation was shown as the plus direction of the error bar; + and Δ represented a significant difference compared to the absorbance of Au-coated cover glasses (Au) + medium and uncoated cover glasses (GL) + medium, respectively; Λ, ∇, and ∴ represented a significant decrease or increase compared to the metabolism levels of the cells cultured on Au, GL, and cell culture-treated polystyrene multi-well culture plates (PS), respectively).

For the MTT assay of NG108-15 neuronal cell line (Figure 3 and Table 3), the absorbance of material + cells was higher than that of corresponding material + medium. The cells cultured on ZnO showed a metabolism level of 5.10% of the baseline, whereas the cells cultured on Au and GL showed metabolism levels of 81.61% and 61.20% of the baseline, respectively.

A two-factor analysis of variance (ANOVA) test with replication (*n* = 3) showed statistical evidence to suggest that substrate material and existence of cells had significant effects on absorbance. For comparisons among the absorbance of substrates with culture medium only, an F-test indicated that there were significant differences between the variances of the absorbance of Au + medium and PS + medium, and GL + medium and PS + medium, and that there were no significant differences between the variances of the absorbance of every other two groups. Thus, a corresponding two-tailed t-test with unequal variances or equal variances indicated that there were significant differences between the absorbance of ZnO + medium and Au + medium, and ZnO + medium and GL + medium. To compare the metabolism of NG108-15 cells cultured on different substrate materials, a single-factor ANOVA showed a significant effect of substrate material on metabolism. A Tukey’s test indicated that the metabolism level of the cells cultured on ZnO showed a significant decrease compared to those of the cells cultured on Au, GL, and PS, that the metabolism levels of the cells cultured on Au and GL showed a significant decrease compared to that of the cells cultured on PS, and that the metabolism level of cells cultured on Au showed a significant increase compared to that of the cells cultured on GL.

### 2.5. MTT Assay of HL-1 Cells

**Figure 4:**
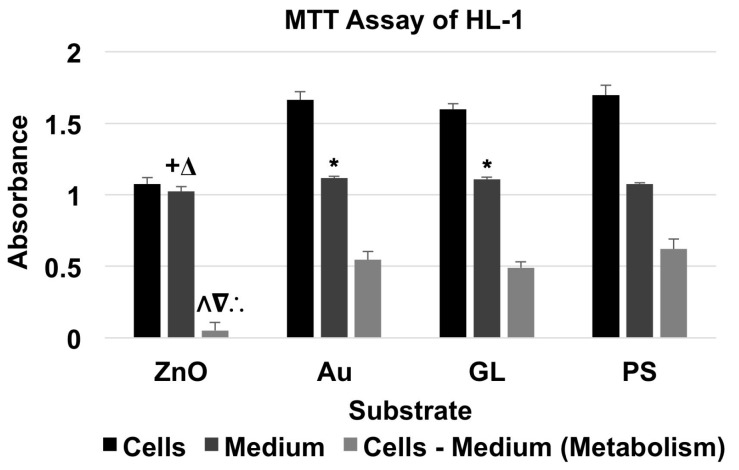
MTT assay of HL-1 cells showed the absorbance of material + HL-1 cells and material + medium, and the absorbance for the metabolism of HL-1 cells (data were presented as mean + standard deviation; standard deviation was shown as the plus direction of the error bar; +, Δ, and * represented a significant difference compared to the absorbance of Au-coated cover glasses (Au) + medium, uncoated cover glasses (GL) + medium, and cell culture-treated polystyrene multi-well culture plates (PS) + medium, respectively; Λ, ∇, and ∴ represented a significant decrease compared to the metabolism levels of the cells cultured on Au, GL, and PS, respectively).

For the MTT assay of HL-1 cardiac muscle cell line (Figure 4 and Table 4), the absorbance of material + cells was higher than that of corresponding material + medium. The cells cultured on ZnO showed a metabolism level of 8.34% of the baseline, whereas the cells cultured on Au and GL showed metabolism levels of 88.05% and 78.95% of the baseline, respectively.

A two-factor ANOVA test with replication (*n* = 3) showed statistical evidence to suggest that substrate material and existence of cells had significant effects on absorbance. For comparisons among the absorbance of substrates with culture medium only, an F-test indicated that there were no significant differences between the variances of the absorbance of every two groups. Thus, a two-tailed t-test with equal variances indicated that there were significant differences between the absorbance of ZnO + medium and Au + medium, ZnO + medium and GL + medium, Au + medium and PS + medium, and GL + medium and PS + medium. To compare the metabolism of HL-1 cells cultured on different substrate materials, a single-factor ANOVA showed a significant effect of substrate material on metabolism. A Tukey’s test indicated that the metabolism level of the cells cultured on ZnO showed a significant decrease compared to those of the cells cultured on Au, GL, and PS.

### 2.6. MTT Assay of Neonatal Cardiomyocytes

**Figure 5:**
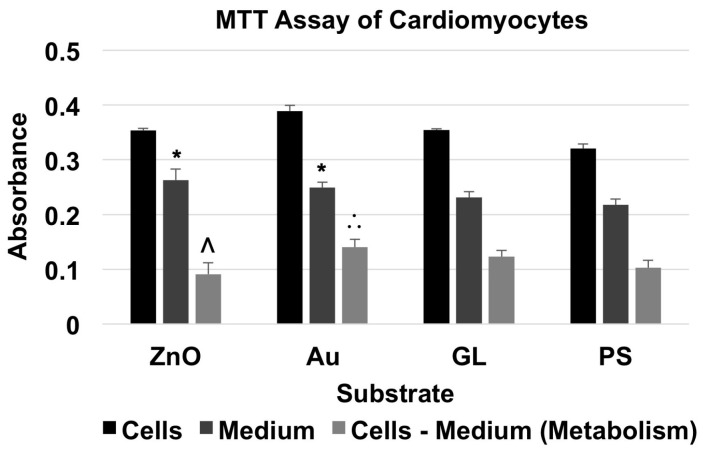
MTT assay of cardiomyocytes showed the absorbance of material + cardiomyocytes and material + medium, and the absorbance for the metabolism of cardiomyocytes (data were presented as mean + standard deviation; standard deviation was shown as the plus direction of the error bar; * represented a significant difference compared to the absorbance of cell culture-treated polystyrene multi-well culture plates (PS) + medium; Λ and ∴ represented a significant decrease or increase compared to the metabolism levels of the cells cultured on Au-coated cover glasses (Au) and PS, respectively).

For the MTT assay of neonatal cardiomyocytes (Figure 5 and Table 5), the absorbance of material + cells was higher than that of corresponding material + medium. The cells cultured on ZnO showed a metabolism level of 88.63% of the baseline, whereas the cells cultured on Au and GL showed metabolism levels of 136.46% and 119.79% of the baseline, respectively.

A two-factor ANOVA test with replication (*n* = 3) showed statistical evidence to suggest that substrate material and existence of cells had significant effects on absorbance. For comparisons among the absorbance of substrates with culture medium only, an F-test indicated that there were no significant differences between the variances of the absorbance of every two groups. Thus, a two-tailed t-test with equal variances indicated that there were significant differences between the absorbance of ZnO + medium and PS + medium, and Au + medium and PS + medium. To compare the metabolism of cardiomyocytes cultured on different substrate materials, a single-factor ANOVA showed that substrate material had a significant effect on metabolism. A Tukey’s test indicated that the metabolism level of the cells cultured on ZnO showed a significant decrease compared to that of the cells cultured on Au, and that the metabolism level of the cells cultured on Au showed a significant increase compared to that of the cells cultured on PS.

## 3. Discussion

**Figure 6:**
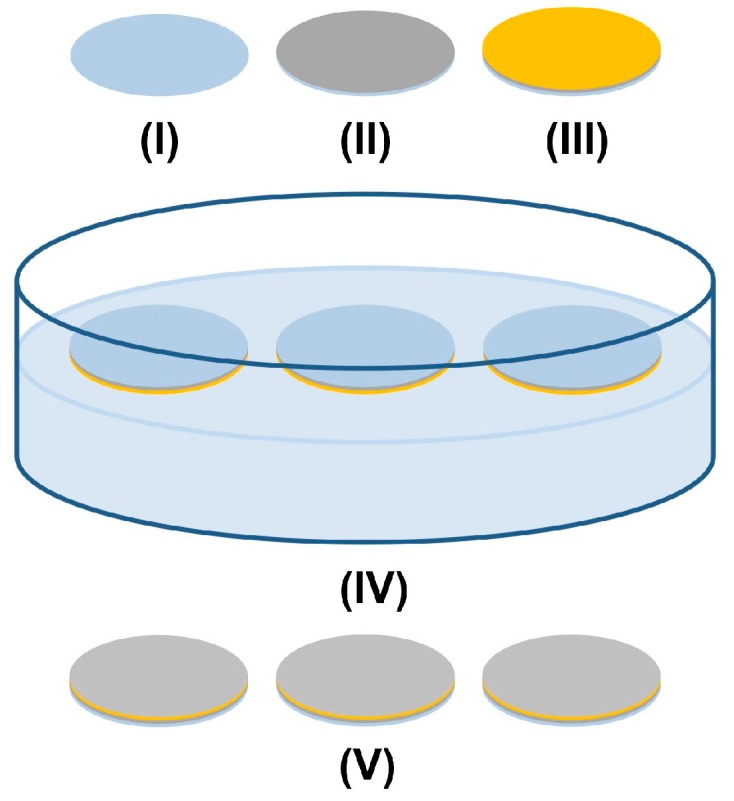
Schematic of synthesis of ZnO nanowire arrays using a seedless solution-based method.

ZnO nanowire arrays as characterized by the SEM images (Figure 1) and XPS data (Figure 2 and Table 1) were synthesized using a seedless solution-based method (Figure 6), which is a versatile way to grow vertically aligned and densely packed ZnO nanowires [23,24]. NG108-15 cells, HL-1 cells, and cardiomyocytes usually have a diameter of around 10–100, 20, and 13 μm, respectively [25,26], Compared to the dimensions (width of ~300 nm) and distribution of the ZnO nanowires shown in Figure 1, each single cell should cover plenty of nanowires when cultured on the nanowire arrays.

The biological effects of ZnO nanowire arrays were tested by measuring the mitochondrial activities of the cells cultured on the nanowire arrays using the MTT assays [27]. Yellow colored MTT was reduced by the mitochondria of living cells (rather than dead cells) into purple colored formazan, which could be dissolved into certain solvents to form a homogeneous solution for colorimetric measurement. The absorbance of the solution indicated the amount of the formed formazan and therefore the cellular metabolism.

For the MTT assays, acidic isopropanol and dimethyl sulfoxide (DMSO) are the two most common solvents used to dissolve formazan [27,28]. DMSO was used in this study instead of the acidic isopropanol. One reason for this was that DMSO enabled colorimetric reading to be completed on the same experiment day without overnight incubation of the solution, as is required by acidic isopropanol. More importantly, DMSO did not dissolve ZnO nanowires, while the metal oxide nanowires were readily dissolved in the acidic isopropanol.

Interestingly, substrate material showed a significant effect on the absorbance of material + medium for all three types of culture media, making the direct comparisons of the absorbance of material + cells within each cell type meaningless. Therefore, for the MTT assays of all three types of cells, a full factorial experiment design was adopted, and the metabolism of cells was evaluated by the net absorbance, calculated by subtracting the absorbance of material + medium from the absorbance of corresponding material + cells.

The MTT assay results showed that ZnO nanowire arrays induced inhibitory effects on all three types of excitable cells after one day in culture. The metabolism level of the NG108-15 cells cultured on ZnO was 5.10% of that of the cells cultured on PS, which was significantly lower than those of the cells cultured on Au, GL, and PS; the metabolism level of the HL-1 cells cultured on ZnO was 8.34% of that of the cells cultured on PS, which was significantly lower than those of the cells cultured on Au, GL, and PS; the metabolism level of the cardiomyocytes cultured on ZnO was 88.63% of that of the cells cultured on PS, which was significantly lower than that of the cells cultured on Au but not significantly different from those of the cells cultured on GL and PS.

The inhibitory effects could be due to the cell membrane penetration of nanowires and/or decreased cell adhesion [29]. The effect of the cell membrane penetration of nanowires particularly depends on the diameter of nanowires. The nanowires in this work had a width of around 300 nm, and nanowires with a comparable diameter of approximately 400 nm induced cell death in mouse embryonic stem cells within one day [30]. Additionally, the cell adhesion could be reduced due to the topography of ZnO nanowire arrays, resulting in the inhibitory effects [31]. Cell adhesion on nanowire arrays is highly dependent on the density and spacing of the nanowires [32]. The high density of nanowires and plentiful spacing (Figure 1b) could result in insufficient planar surface and binding sites for focal adhesion, leading to reduced cell adhesion [29,32].

Other than the physical factors, the chemistry of ZnO nanowires could also induce cytotoxicity similarly to ZnO nanoparticles mainly via the overproduction of reactive oxygen species and/or release of free Zn^2+^ ions [33,34,35]. The overproduction of reactive oxygen species could cause excessive oxidative stress and oxidant injury in cells [36]. As zinc oxide nanowires were biodegradable in biological fluids [37], the cytotoxicity could also be due to the excess of released zinc ions resulting from the extracellular degradation of ZnO nanowires [38]. Cells could also phagocytize ZnO nanowires, and the zinc ions released from the intracellular degradation of the internalized ZnO nanowires in the acidic environment of lysosome could also induce cytotoxicity [12]. However, it would be more difficult for cells to phagocytize ZnO nanowires from the immobilized arrays compared to the disperse nanowires.

Interestingly, after one day in culture, the metabolism levels of NG108-15 and HL-1 cells decreased to 5.10% and 8.34% of their corresponding baselines, respectively, while the metabolism level of primary cardiomyocytes only decreased to 88.63% of the baseline. Also, NG108-15 and HL-1 cells cultured on ZnO nanowire arrays both showed a significant decrease in metabolism compared to the ones cultured on gold, glass, and polystyrene substrates, whereas primary cardiomyocytes cultured on ZnO nanowire arrays only showed a significant decrease in metabolism compared to the ones cultured on gold substrate, which showed a significant increase in metabolism compared to the ones cultured on polystyrene substrate. Primary cardiomyocytes seemed to better tolerate the inhibitory effects induced by ZnO nanowire arrays, which was consistent with previous studies showing that ZnO nanoparticles induced selectively higher cytotoxicity on rapidly dividing cell lines than on primary cells [39,40,41,42,43]. The cytotoxicity of ZnO nanomaterials depends on multiple factors, such as cell type, the size of nanomaterial, the shape of nanomaterial, culture condition, etc. [35]. The difference in the inhibitory effects on NG108-15 cells, HL-1 cells, and primary cardiomyocytes could also be due to differences in the serums and phosphates in the culture media and in the proteins used to pre-coat the substrates [44,45,46].

Although biocompatibility can be improved by decreasing the amount of ZnO nanowires, it would be difficult to do so for vertically aligned and densely packed high aspect ratio ZnO nanowire arrays, as most of the given surface area was occupied by nanowires in spite of their dimensions. However, varying the dimensions and spacing of ZnO nanowires could change the topography of the arrays and therefore could affect the interactions between the substrate and cells. The extracellular degradation of nanowires and release of free zinc ions would be another challenge to make the ZnO nanowire arrays biocompatible for long-term applications. Thus, the mechanism of these inhibitory effects on excitable cells needs to be further addressed in future research, as this is critical for the biomedical applications of ZnO nanowire arrays.

## 4. Materials and Methods 

### 4.1. Materials

Zinc nitrate hexahydrate, hexamethylenetetramine, ammonium hydroxide solution (28.0%–30.0% NH_3_ basis), sodium bicarbonate (NaHCO_3_), sodium chloride (NaCl), potassium chloride (KCl), sodium phosphate dibasic (Na_2_HPO_4_), magnesium sulfate (MgSO_4_), Hepes, glucose, Claycomb medium, fetal bovine serum (FBS) for HL-1 cells, norepinephrine, l-glutamine, gelatin, fibronectin, pancreatin, and 3-(4,5-dimethyl-2-thiazolyl)-2,5-diphenyl-2*H*-tetrazolium bromide (MTT) were purchased from Sigma-Aldrich (Saint Louis, MO, USA). Dulbecco’s modified eagle’s medium (DMEM) without sodium pyruvate, DMEM, distilled water, phosphate-buffered saline (PBS, pH 7.4), penicillin-streptomycin, hypoxanthine-aminopterin-thymidine, collagenase type II, newborn calf serum (NCS), horse serum, and charcoal-stripped FBS for neonatal cardiomyocytes were purchased from Thermo Fisher Scientific (Waltham, NA, USA). Non-heat-inactivated FBS for NG108-15 cells was purchased from GE Healthcare Life Sciences (Pittsburgh, PA, USA). Circular cover glasses of 18 mm diameter were purchased from Fisher Scientific (Hampton, NH, USA).

### 4.2. Synthesis of ZnO Nanowire Arrays

ZnO nanowire arrays were synthesized via a solution-based method as shown in Figure 6 [23,24]. Circular cover glasses of 18 mm diameter were washed in sequence in acetone, 2-propanol, and distilled water in an ultrasonic bath for 5 min in each solvent. The cover glasses were thereafter dried with an air gun and baked on a 200 °C hot plate for 5 min to remove remaining moisture (I). A 10 nm thick layer of titanium (II) and a 50 nm thick layer of gold (III) were sequentially deposited onto the cover glasses using an e-beam evaporator (DV-502A, Denton Vacuum, Moorestown, NJ, USA). The coating was annealed in a 300 °C furnace for 1 h. With the gold coating in contact with the solution, three coated cover glasses were gently placed in a Petri dish to float on the top of 40 mL of aqueous solution containing 25 mM zinc nitrate hexahydrate, 12.5 mM hexamethylenetetramine, and 0.70 M ammonium hydroxide in an 80 °C oven (Gravity Ovens, Fisher Scientific, Hampton, NH, USA) for 24 h (IV). The lid of the Petri dish was kept on during the entire reaction. The ZnO nanowire arrays (V) grown on the gold-coated surface were washed thoroughly with distilled water and air dried.

### 4.3. SEM

The morphological features of the ZnO nanowire arrays were imaged by a scanning electron microscope (Nova NanoSEM 400, FEI, Hillsboro, OR, USA) using an Everhart-Thornley detector in high vacuum mode. No coating was applied. Images were processed and analyzed using ImageJ (Research Services Branch, NIH, Bethesda, MD, USA).

### 4.4. XPS

The surface chemistry of the ZnO nanowire arrays was analyzed by an X-ray photoelectron spectroscopy (Axis Ultra, Kratos Analytical, Manchester, UK) using a monochromatic A1 Kα X-ray source operated at 12 kV and 10 mA. The XPS data were processed and analyzed using CasaXPS (Casa Software Ltd., Devon, UK).

### 4.5. Cell Culture

NG108-15 glioblastoma and neuroblastoma hybrid cell line was purchased from ATCC (Manassas, VA, USA). NG108-15 cells were cultured in DMEM without sodium pyruvate supplemented with 10% non-heat-inactivated FBS, 100 U/mL penicillin, 100 μg/mL streptomycin, 0.1 mM hypoxanthine, 400 nM aminopterin, 0.016 mM thymidine, and 1.5 g/L NaHCO_3_ under 5% carbon dioxide (CO_2_) and 95% air at 37 °C.

HL-1 cardiac muscle cell line was a courtesy gift from William C. Claycomb at Louisiana State University Health New Orleans, New Orleans, LA, USA. HL-1 cells were cultured in Claycomb medium supplemented with 10% FBS, 100 U/mL penicillin, 100 μg/mL streptomycin, 0.1 mM norepinephrine, and 2 mM l-glutamine in the gelatin (200 μg/mL) and fibronectin (5 μg/mL) pre-coated flasks under 5% CO_2_ and 95% air at 37 °C.

Neonatal cardiomyocytes were harvested from 2 days old Sprague-Dawley rat pups according to a protocol by Richard Pattern at Tufts Medical Center, Boston, MA, USA [47]. Briefly, hearts from eleven neonatal Sprague-Dawley rat pups were surgically removed and temporarily immersed in a dissociation buffer solution (116 mM NaCl, 5.4 mM KCl, 0.8 mM Na_2_HPO_4_, 0.8 mM MgSO_4_, 20 mM Hepes, and 5.6 mM glucose, pH 7.35). After the extraction of all the hearts, tissue was transferred into an enzyme buffer solution (dissociation buffer solution with 0.06% pancreatin and 0.04% collagenase type II) and was finely chopped into pieces. The tissue suspension was placed in a 37 °C shaker at approximately 80–86 rpm for serial digestion. Four rounds of digestion were performed for 5 min, 20 min, 25 min, and 25 min, respectively. After each digestion, the cell suspension was placed immediately in 2 mL of NCS and then centrifuged at 100 G for 5 min. The resultant pellet was then re-suspended in 4 mL of NCS and kept at 37 °C in the incubator. After all digestions, all the cell suspensions in NCS were mixed together and centrifuged again at 100 G for 5 min, and then the pellet was re-suspended in 4 mL of culture medium (DMEM supplemented with 10% horse serum, 7% charcoal-stripped FBS, 100 U/mL penicillin, and 100 μg/mL streptomycin). To remove non-dissociated tissue, the suspension was passed through a 70 μm nylon filter. Finally, to remove fibroblasts, a 75-min pre-plating procedure was conducted by plating the suspension on two 60 mm^2^ Petri dishes at 37 °C in the incubator. The cell suspension was ready for counting and plating after pre-plating. The cardiomyocytes were cultured in the culture medium in the fibronectin (25 μg/mL) pre-coated flasks under 5% CO_2_ and 95% air at 37 °C. Animal experiments followed protocol approved by the Institutional Animal Care and Use Committee at The Ohio State University.

### 4.6. MTT Assays

MTT assays were conducted following the standard protocol [27,28]. ZnO nanowire arrays, Au-coated cover glasses, and uncoated cover glasses were first sterilized by being immersed in an aqueous 70% ethanol solution for 30 min and then thoroughly washed with PBS three times. Each substrate was placed into a well of a 12-well microplate with the nanowire arrays and Au coating facing up. The polystyrene wells of the microplate served as another substrate. All groups were repeated in triplicate (*n* = 3).

Before plating HL-1 cells, all substrates were pre-coated with gelatin (200 μg/mL) and fibronectin (5 μg/mL) for 1 h, and before plating neonatal cardiomyocytes, all substrates were pre-coated with fibronectin (25 μg/mL) overnight. 1 mL of cell suspension of NG108-15 cells, HL-1 cells, or neonatal cardiomyocytes at a cell density of 1 × 10^5^ cells/mL was plated into each well. After the cells were incubated under 5% CO_2_ and 95% air at 37 °C for 20 h, the culture medium was replaced with another 1 mL of fresh culture medium, and 100 μL of sterile filtered 0.5% MTT in PBS was added to each well. After another 4-h incubation, 750 μL of culture medium was removed, and 1 mL of sterile filtered DMSO was added to each well to dissolve the formazan. Until the formazan was observed to be fully dissolved under the microscope forming homogeneous solutions, each solution was transferred to a 96-well microplate for reading. The absorbance was read at the wavelengths of 540 nm and 690 nm using a microplate reader (FlexStation 3, Molecular Devices, Sunnyvale, CA, USA). The absorbance at 690 nm served as a reference and was subtracted from the absorbance at 540 nm.

## 5. Conclusions

The biological effects of nanomaterials can depend on a variety of factors, including their physiochemical attributes and the type of the cells they interact with. Thus, their biosafety needs to be assessed under the settings for a specific application. This work studied the biological effects of vertically aligned and densely packed high aspect ratio ZnO nanowire arrays on excitable cells cultured on top and revealed that the ZnO nanowire arrays caused inhibitory effects on a neuronal cell line and a cardiac muscle cell line, whereas primary neonatal rat cardiomyocytes showed better tolerance to the inhibitory effects. Therefore, for the biomedical applications of ZnO nanowire arrays, particularly to interact with the nervous system or heart, their biosafety needs to be specifically studied and addressed, with inhibitory effects to be more likely.

## Figures and Tables

**Table 1:** Percentage atomic concentrations of regions and components detected on the surface of ZnO nanowire arrays by X-ray photoelectron spectroscopy (XPS).

Regions and Components	Percentage Atomic Concentration (%)
Zn 2p	19.51
O 1s (531.7 eV)	20.58
O 1s (530.2 eV)	20.92
C 1s	38.99

**Table 2:** MTT assays of a full factorial 2 × 4 design (ZnO nanowire arrays (ZnO), Au-coated cover glasses (Au), uncoated cover glasses (GL), and cell culture-treated polystyrene multi-well culture plates (PS)).

	ZnO	Au	GL	PS
Cells	ZnO + Cells	Au + Cells	GL + Cells	PS + Cells
Medium	ZnO + Medium	Au + Medium	GL + Medium	PS + Medium

**Table 3:** The absorbance of the MTT assay of NG108-15 cells.

		ZnO	Au	GL	PS
Cells	Mean	0.1783	0.784389	0.619722	0.9331
	SD	0.002201	0.072758	0.07081	0.030728
Medium	Mean	0.137311	0.128267	0.1277	0.129122
	SD	0.001835	0.001212	0.00145	0.006888
Metabolism	Mean ^1^	0.040989	0.656122	0.492022	0.803978
	SD ^2^	0.002865	0.072768	0.070825	0.031491

^1^ The mean value (mean) of the absorbance for metabolism was calculated by subtracting the absorbance of the corresponding material + medium from the absorbance of the corresponding material + cells; ^2^ The standard deviation (SD) of the absorbance for metabolism was calculated by extracting the square root of the sum of squares of the standard deviations of the corresponding material + cells and material + medium.

**Table 4:** The absorbance of the MTT assay of HL-1 cells.

		ZnO	Au	GL	PS
Cells	Mean	1.075189	1.663778	1.598511	1.696133
	SD	0.043464	0.0558	0.039735	0.070086
Medium	Mean	1.023422	1.117367	1.108578	1.075533
	SD	0.03335	0.012897	0.014967	0.008013
Metabolism	Mean ^1^	0.051767	0.546411	0.489933	0.6206
	SD ^2^	0.054784	0.057271	0.04246	0.070542

^1,2^ The mean and SD of the absorbance for metabolism were calculated by using the same methods described in the footers of Table 3.

**Table 5:** The absorbance of the MTT assay of cardiomyocytes.

		ZnO	Au	GL	PS
Cells	Mean	0.353844	0.389156	0.354022	0.320267
	SD	0.003833	0.010207	0.002787	0.008732
Medium	Mean	0.262844	0.249044	0.231022	0.217589
	SD	0.020086	0.010065	0.010703	0.010902
Metabolism	Mean ^1^	0.091	0.140111	0.123	0.102678
	SD ^2^	0.020449	0.014335	0.01106	0.013968

^1,2^ The mean and SD of the absorbance for metabolism were calculated by using the same methods described in the footers of Table 3.
